# Central executive network-related neuromagnetic abnormalities in chronic and episodic migraine: a resting-state magnetoencephalography study

**DOI:** 10.3389/fnins.2026.1865928

**Published:** 2026-09-01

**Authors:** Lin Huang, Xu Huang, Yuanwen Yu, Chuanyong Yu, Yu Wang

**Affiliations:** 1 Department of Neurology, Epilepsy and Headache Group, The First Affiliated Hospital of Anhui Medical University, Hefei, China; 2 Department of Neurology, The Affiliated Brain Hospital of Nanjing Medical University, Nanjing, Jiangsu, China; 3 Department of Neurology, Anhui Public Health Clinical Center, Hefei, China

**Keywords:** central executive network, chronic migraine, episodic migraine, functional connectivity, magnetoencephalography, spectral power

## Abstract

**Background:**

Chronic migraine (CM) is more disabling than episodic migraine (EM), but the neurophysiological mechanisms underlying migraine chronification remain incompletely understood. We investigated central executive network (CEN)-related neuromagnetic abnormalities in CM and EM.

**Methods:**

The original cohort included 22 patients with CM, 20 with EM, and 25 healthy controls (HCs). An independent validation cohort included 30 patients with CM and 30 with EM. Source-space analyses focused on 10 predefined CEN-related regions from the Desikan–Killiany atlas. Relative power spectral density (PSD) across six frequency bands and functional connectivity using corrected amplitude envelope correlation and network-based statistics were assessed. An exploratory support vector machine (SVM) analysis evaluated the discriminative value of CEN-related spectral and connectivity features.

**Results:**

PSD abnormalities were mainly observed in the theta, beta, and gamma1 bands and were largely driven by CM. Compared with HCs, CM showed increased theta-band relative power in left frontal CEN-related regions and reduced beta- and gamma1-band relative power in right frontal regions. Compared with EM, CM showed increased theta-band relative power in left frontal regions. Functional connectivity analysis revealed frequency-specific subnetworks in the theta, beta, delta, and gamma1 bands. Compared with HCs, CM showed increased theta- and gamma1-band connectivity and reduced beta-band connectivity, whereas EM showed more limited reductions in beta- and gamma1-band connectivity. CM also showed stronger delta- and gamma1-band connectivity than EM, predominantly involving prefrontal regions. The SVM combining two PSD and eight connectivity features achieved an accuracy of 90.48% and an AUC of 0.9773 (95% CI: 0.93–1.00) in the original cohort, and an accuracy of 86.67% and an AUC of 0.8322 (95% CI: 0.76–0.95) in the independent validation cohort.

**Conclusion:**

CM is associated with more extensive and frequency-specific CEN-related neuromagnetic abnormalities than EM, particularly within prefrontal–frontoparietal circuits. These findings suggest altered executive-control-related cortical dynamics during migraine chronification, while combined spectral and connectivity features may help differentiate CM from EM.

## 1 Introduction

Migraine is one of the most common and disabling neurological disorders worldwide and remains a major public health challenge (Steiner et al., 2024). Within the migraine spectrum, chronic migraine (CM) represents a more severe clinical phenotype than episodic migraine (EM), and is associated with greater functional disability, poorer quality of life, and higher healthcare utilization (Blumenfeld et al., 2011; Stokes et al., 2011; Shao et al., 2022). However, although migraine is defined by relatively well-established clinical diagnostic criteria, the neural mechanisms underlying migraine chronification remain incompletely understood, and objective neurophysiological signatures that may help characterize disease progression are still lacking (May and Schulte, 2016; Lipton et al., 2023).

Accumulating evidence suggests that migraine should not be viewed merely as a recurrent pain disorder, but rather as a large-scale brain network disorder involving sensory, affective, and cognitive systems (Coppola et al., 2019; Pozo-Rosich et al., 2021; Schramm et al., 2023). From this perspective, executive-control-related networks are of particular interest because they play key roles in cognitive control, attentional allocation, and the top-down regulation of behaviorally relevant information (Power et al., 2011; Cocuzza et al., 2020). Previous resting-state functional magnetic resonance imaging (fMRI) studies have reported impaired functional connectivity between the frontoparietal network and posterior associative cortices such as the precuneus in patients with migraine (Li et al., 2017). Subsequently, Androulakis et al. further demonstrated in women with chronic migraine that resting-state connectivity was reduced in the central executive network (CEN), default mode network (DMN), and salience network (SN), and that greater headache burden was associated with lower CEN connectivity (Androulakis et al., 2017). These findings suggest that dysfunction in executive-control-related brain networks may represent an important component of migraine chronification rather than a secondary consequence of pain alone.

Despite these advances, electrophysiological evidence specifically addressing CEN-related dysfunction in migraine remains limited. Magnetoencephalography (MEG) is well suited to investigate this question because it directly records neuronal activity with millisecond temporal resolution and enables frequency-specific characterization of both local oscillatory activity and interregional functional coupling at the source-space level (Baillet, 2017). Compared with fMRI, MEG is more sensitive to transient electrophysiological dynamics; compared with electroencephalography (EEG), it provides superior cortical source localization (Hall et al., 2014; Hedrich et al., 2017). Previous MEG studies have shown frequency-dependent abnormalities in resting-state brain activity and connectivity in migraine (Hsiao et al., 2021; Zebhauser et al., 2024). For example, Zhang et al., in a study focusing on the DMN, reported that alpha-band power in the left medial prefrontal cortex was lower in CM than in EM and was negatively correlated with headache frequency (Zhang et al., 2024). Hsiao et al. found abnormalities in temporal stability and neural complexity within sensory-cognitive networks in patients with migraine and further showed that relevant MEG-derived features could distinguish CM from EM with good classification accuracy (Hsiao et al., 2022, 2025). Nevertheless, source-space MEG studies directly comparing CM and EM within predefined CEN-related regions, while simultaneously assessing local spectral activity and within-network functional connectivity, remain scarce.

Therefore, in the present resting-state MEG study, we compared patients with CM, EM, and healthy controls to investigate neuromagnetic abnormalities in CEN-related regions in migraine without aura. Focusing on 10 predefined CEN-related regions of interest derived from the Desikan–Killiany atlas, we examined relative spectral power across six frequency bands and assessed source-space functional connectivity using corrected amplitude envelope correlation and network-based statistics. In addition, we performed an exploratory support vector machine analysis to evaluate the potential discriminative value of CEN-related spectral and connectivity features for distinguishing CM from EM. Specifically, we aimed to determine whether migraine chronification is associated with more extensive and frequency-specific alterations in executive-control-related cortical dynamics during the interictal state.

## 2 Materials and methods

### 2.1 Subjects

Patients in the original cohort were recruited from the Headache Clinic of the Department of Neurology, the Affiliated Brain Hospital of Nanjing Medical University between November 2024 and December 2025. HCs were recruited from the local community during the same period and were matched to the patient groups for age and sex. All patients were screened and diagnosed by neurologists with specialized training in headache disorders according to the International Classification of Headache Disorders, 3rd edition (ICHD-3, 2018). Based on headache frequency, patients were classified as having EM or CM. EM was defined as fewer than 15 headache days per month, whereas CM was defined as at least 15 headache days per month for more than 3 months, with migraine features present on at least 8 days per month.

The original cohort comprised 67 participants, including 42 patients with MwoA (22 CM and 20 EM) and 25 HCs. To independently validate the SVM classifier, an additional independent validation cohort comprising 60 patients with MwoA (30 CM and 30 EM) was subsequently enrolled at the same center between January and July 2026. These participants were enrolled after completion of recruitment for the original cohort and were used exclusively for independent validation. Patients in the independent validation cohort met the same diagnostic and eligibility criteria as those in the original cohort, with no participant overlap between the two cohorts.

All participants were right-handed and aged 18–60 years. None of the migraine patients had received prophylactic treatment for migraine or had a history of medication overuse. The exclusion criteria were as follows: other neurological disorders, psychiatric symptoms, current use of medications affecting the central nervous system, contraindications to MEG, inability to cooperate with the examination, and inability to complete MRI because of claustrophobia or other reasons. HCs had no history of headache or neurological disease and no family history of migraine in first-degree relatives.

This study was approved by the Medical Ethics Committee of Nanjing Brain Hospital (approval number: 2022-KY063-01). Written informed consent was obtained from all participants before enrollment.

### 2.2 Study design

This was a cross-sectional case–control study designed to compare resting-state MEG characteristics among patients with CM, patients with EM, and HCs. All clinical data were collected by trained neurology professionals before MEG and MRI acquisition, including sex, age, disease duration, monthly headache frequency, pain intensity assessed using the visual analog scale (VAS), and duration of individual attacks. Anxiety and depressive symptoms were assessed in all participants using the Hamilton Anxiety Rating Scale (HAMA) and the Hamilton Depression Rating Scale (HAMD), respectively.

All migraine patients underwent resting-state MEG recording during the interictal period. The interictal period was defined as the absence of a migraine attack for at least 72 h before MEG acquisition and no recurrence within 24 h after the recording. If these criteria were not met, the MEG examination was rescheduled. All participants completed clinical assessment, MRI examination, and MEG acquisition according to a standardized study protocol.

### 2.3 Data acquisition

#### 2.3.1 MEG acquisition

MEG data were acquired in a magnetically shielded room at the MEG Center of Nanjing Brain Hospital using a whole-head CTF 275-channel MEG system (CTF Systems Inc., Coquitlam, BC, Canada). Before acquisition, all participants were instructed to remove metallic objects to avoid interference with magnetic signals. Three localization coils were placed at the nasion and bilateral preauricular points to determine head position relative to the MEG sensors and to enable subsequent co-registration of MEG and MRI data. Head position was continuously monitored during acquisition. If head movement exceeded 5 mm in any recording segment, that segment was discarded and reacquired.

MEG data were sampled at 6000 Hz, and third-order gradient noise cancellation was applied to reduce environmental noise. No online temporal band-pass, low-pass, or high-pass filtering was applied during MEG acquisition. All participants underwent resting-state recording in a supine position while awake, relaxed, and with their eyes closed. Six consecutive resting-state recordings were obtained from each participant, each lasting 120 s. If the participant fell asleep during acquisition or if the data contained obvious motion artifacts, that segment was excluded from further analysis and reacquired when necessary. Before formal acquisition, a 3-min empty-room recording was performed to capture environmental and sensor noise, which was later used to estimate the noise covariance matrix for source analysis. Finally, one continuous 30-s artifact-free resting-state segment was selected from the retained data for subsequent analyses (da Silva Castanheira et al., 2021).

#### 2.3.2 MRI acquisition

All participants underwent structural MRI scanning on a 3.0 T scanner (Siemens, Munich, Germany). High-resolution three-dimensional T1-weighted images were acquired using an MPRAGE sequence with the following parameters: TR/TE = 1900/2.48 ms, FOV = 250 × 250 mm, flip angle = 9°, slice thickness = 1 mm, voxel size = 0.48 × 0.48 × 1 mm^3^, and matrix size = 512 × 512. The nasion and bilateral preauricular points were used as common anatomical landmarks for co-registration of MEG and MRI data to determine the anatomical localization of source activity.

### 2.4 Data processing

#### 2.4.1 Preprocessing and source reconstruction

All offline MEG preprocessing, source reconstruction, and subsequent source-level analyses were performed using Brainstorm (Tadel et al., 2011), following its published resting-state MEG processing framework. The CTF system and manufacturer-provided software were used only for data acquisition and online third-order spatial gradient noise cancellation and were not involved in any offline preprocessing or source-level analysis. Structural MRI data were processed using FreeSurfer for cortical surface reconstruction (Fischl, 2012). To reduce the effects of environmental noise and non-neural physiological artifacts, raw MEG data were first visually inspected to identify and exclude segments contaminated by marked head motion, transient noise, or other abnormal interference. A notch filter at 50 Hz and its harmonics was then applied to remove power-line contamination. Beyond the notch filter, no other temporal filtering was applied to the broadband sensor-level data during offline preprocessing. Ocular and cardiac artifacts were further identified using simultaneously recorded electrooculogram (EOG) and electrocardiogram (ECG) signals and corrected using the signal-space projection (SSP) method (Niso et al., 2019). All subsequent analyses were based on a continuous 30-s artifact-free resting-state segment extracted from each participant.

Source-space analyses were performed across six frequency bands: delta (2–4 Hz), theta (5–7 Hz), alpha (8–12 Hz), beta (15–29 Hz), gamma1 (30–59 Hz), and gamma2 (60–90 Hz).

To estimate cortical source activity from sensor-level MEG data, we used a depth-weighted minimum-norm estimation (MNE) distributed source model (Gramfort et al., 2014). Individual T1-weighted structural images were automatically reconstructed in Free Surfer to obtain cortical surface models and gray/white matter boundary information. A forward model was then constructed in Brainstorm based on each individual MRI using the overlapping spheres method, with the cortical surface represented by approximately 15,000 vertices. The inverse solution was computed under the following constraints: source orientations were constrained to be perpendicular to the cortical surface; depth weighting was applied to reduce the inherent bias of MNE toward superficial sources; and the regularization parameter was set to λ^2^ = 0.33, corresponding to an SNR of 3, to reduce numerical instability and noise sensitivity. Subsequent spectral power and functional connectivity analyses in each frequency band were performed in source space.

Cortical parcellation was based on the Desikan-Killiany atlas (Desikan et al., 2006). Based on previous studies of the CEN and the design of the present study, 10 CEN-related ROIs were selected for further analysis, including the bilateral caudal middle frontal gyrus (Caudal MFG-L/R), bilateral rostral middle frontal gyrus (Rostral MFG-L/R), bilateral superior frontal gyrus (SFG-L/R), bilateral inferior parietal lobule (IPL-L/R), and bilateral precuneus (PCu-L/R) (Power et al., 2011).

#### 2.4.2 MEG data analysis

##### 2.4.2.1 CEN spectral power analysis

After source reconstruction, time series were extracted from the 10 CEN-related ROIs in source space, and spectral power analysis was performed at the ROI level. For each ROI, source-level oscillatory activity was estimated using the mean current density across all vertices within that region, and PSD was subsequently calculated. PSD was estimated using Welch's method with a 5-s window and 50% overlap. Power within each frequency band was then calculated and normalized to the total power across all analyzed frequency bands to obtain relative PSD values, thereby facilitating comparisons across regions and participants (Niso et al., 2019).

##### 2.4.2.2 CEN functional connectivity analysis

Oscillatory functional connectivity within the CEN was quantified using AEC-c (Brookes et al., 2011). Depth-weighted MNE source reconstruction was first performed on the preprocessed broadband sensor-level MEG data. The resulting source-space time series were then extracted from each ROI and band-pass filtered into the predefined frequency bands before connectivity estimation. To reduce spurious correlations related to volume conduction and field spread, each signal pair was orthogonalized prior to envelope extraction (Colclough et al., 2015). The amplitude envelope was derived from the absolute value of the Hilbert transform of the band-pass-filtered source activity, reflecting temporal fluctuations in oscillatory amplitude. Functional connectivity strength between two ROIs was defined as the Pearson correlation coefficient between their amplitude envelope time series.

Pairwise connectivity was calculated among the 10 CEN-related ROIs, resulting in a symmetric 10 × 10 functional connectivity matrix for each participant in each frequency band. Higher AEC-c values indicated stronger temporal synchronization of amplitude fluctuations between two brain regions and, therefore, stronger oscillatory coupling (Colclough et al., 2016).

### 2.5 Statistical analysis

All statistical analyses were performed using SPSS (version 25.0; IBM Corp., Armonk, NY, USA), and exploratory machine-learning analyses were conducted in Python. Normality of continuous variables was assessed using the Kolmogorov–Smirnov test. Categorical variables were presented as counts (percentages) and compared using the chi-square test. Continuous variables were expressed as mean ± standard deviation or median (interquartile range), depending on their distribution. Demographic characteristics among the three groups were compared using one-way analysis of variance or the Kruskal–Wallis test. Migraine-related clinical variables were compared only between the CM and EM groups, using the independent-samples t test for normally distributed data and the Mann–Whitney U test for non-normally distributed data. Unless otherwise specified, all tests were two-sided, and P < 0.05 was considered statistically significant.

To determine whether the observed neurophysiological differences were independent of emotional symptoms, HAMA and HAMD scores were included as covariates in the group-level analyses of the MEG-derived measures.

Between-group differences in relative PSD across the 10 CEN-related ROIs and six frequency bands were assessed using analysis of covariance (ANCOVA), with group as the fixed factor and HAMA and HAMD scores as covariates. For each predefined ROI–frequency measure with a significant overall group effect, three covariate-adjusted pairwise comparisons were performed (CM vs. EM, CM vs. HCs, and EM vs. HCs). Bonferroni correction was applied, yielding a corrected significance threshold of *P* < 0.0167.

Differences in CEN functional connectivity were analyzed using network-based statistics (NBS) (Zalesky et al., 2010). The analysis included 45 possible connections among the 10 CEN-related ROIs and was performed separately for each of the six frequency bands. For each pairwise group comparison (CM vs. EM, CM vs. HCs, and EM vs. HCs), an edge-wise general linear model was constructed, with group as the variable of interest and HAMA and HAMD scores included as covariates. A primary threshold of *P* < 0.01 was used to detect exploratory large-scale network effects, whereas a more stringent threshold of *P* < 0.001 was applied to identify more localized effects. Component-level significance was evaluated using 5,000 non-parametric permutations, with the size of the largest connected component in each permutation serving as the test statistic. Statistical significance was determined after family-wise error rate correction, with a corrected threshold of *P* < 0.05.

To further explore the potential discriminative value between CM and EM, an exploratory SVM classification analysis was performed. Candidate features were derived from CEN-related spectral power and functional connectivity measures that remained significant in the covariate-adjusted CM–EM comparisons. Candidate feature selection was performed using the full original CM–EM dataset before cross-validation and was not repeated within each cross-validation fold. The model was implemented in Python using the scikit-learn library with a linear kernel, a penalty parameter of C = 1.0, and class_weight = “balanced.” Feature standardization and model training were performed within a five-fold stratified cross-validation framework, and model performance was evaluated using accuracy, area under the curve (AUC), sensitivity, and specificity. To further assess model generalizability, an independent validation cohort comprising 60 additional patients (30 CM and 30 EM) from the same center was used, with no participant overlap with the original model-development cohort. The validation data were processed using the same preprocessing, source reconstruction, and feature-extraction procedures as those used for the original cohort. The same 10 predefined features listed in Table 1 were extracted, and the finalized classifier trained on the original cohort was applied directly to the independent validation cohort without additional feature selection, model retraining, or hyperparameter optimization. Ninety-five percent confidence intervals for AUC were estimated using 5,000 bootstrap resamples of the out-of-fold predictions in the original cohort and the independent predictions in the validation cohort.

**Table 1:** Features included in the exploratory SVM analysis.

No.	Feature type	Frequency band	Region/ connection	Direction (CM vs. EM)
1	Relative PSD	Theta	Caudal MFG-L	CM > EM
2	Relative PSD	Theta	SFG-L	CM > EM
3	Functional connectivity	Delta	Caudal MFG-L—SFG-L	CM > EM
4	Functional connectivity	Delta	Caudal MFG-R—SFG-L	CM > EM
5	Functional connectivity	Delta	Caudal MFG-R—SFG-R	CM > EM
6	Functional connectivity	Gamma1	Caudal MFG-L—Caudal MFG-R	CM > EM
7	Functional connectivity	Gamma1	Caudal MFG-L—Rostral MFG-L	CM > EM
8	Functional connectivity	Gamma1	Caudal MFG-L—SFG-L	CM > EM
9	Functional connectivity	Gamma1	Rostral MFG-R—SFG-L	CM > EM
10	Functional connectivity	Gamma1	Rostral MFG-R—SFG-R	CM > EM

## 3 Results

### 3.1 Demographic and clinical characteristics

Table 2 summarizes the clinicodemographic characteristics of the original cohort, which included 67 participants: 22 patients with CM, 20 patients with EM, and 25 HCs. No significant between-group differences were observed in age or sex distribution. The demographic and clinical characteristics of the independent validation cohort are summarized in Supplementary Table 1. Among the migraine groups, as expected, the monthly headache frequency was significantly higher in the CM group than in the EM group (20.0 ± 5.5 vs. 5.3 ± 4.1 days/month, *p* < 0.001). In addition, painkiller use was more frequent in the CM group than in the EM group (6.3 ± 2.1 vs. 3.2 ± 1.3 days/month, *p* < 0.001), and VAS scores were also higher in the CM group (7.7 ± 1.6 vs. 6.6 ± 1.6, *p* = 0.031). By contrast, disease duration did not differ significantly between the CM and EM groups (16.5 ± 8.5 vs. 15.8 ± 9.7 years, *p* = 0.817).

**Table 2:** Demographic and clinical characteristics.

Clinical characteristics	CM	EM	HC	*p*-value
Sex	8 M/14 F	7 M/13 F	10 M/15 F	0.936
Age(y)	40.5 ± 11.1	36.9 ± 12.1	34.2 ± 8.5	0.131
Headache frequency (days/ month)	20.0 ± 5.5	5.3 ± 4.1	/	< 0.001^*^
Disease duration (y)	16.5 ± 8.5	15.8 ± 9.7	/	0.817
Painkiller use (days/month)	6.3 ± 2.1	3.2 ± 1.3	/	< 0.001^*^
VAS score	7.7 ± 1.6	6.6 ± 1.6	/	0.031^*^
HAMA score	11.3 ± 6.6	10.7 ± 5.1	2.9 ± 1.8	< 0.001^*^
HAMD score	9.9 ± 4.5	8.7 ± 4.1	2.3 ± 1.5	< 0.001^*^

SD, standard deviation; CM, chronic migraine; EM, episodic migraine; HC, healthy controls; HAMA, hamilton anxiety rating scale; HAMD, hamilton depression rating scale; VAS, visual analog scale; M, male; F, female. ^*^p < 0.05 is statistically significant.

### 3.2 Spectral power analysis

**Figure 1:**
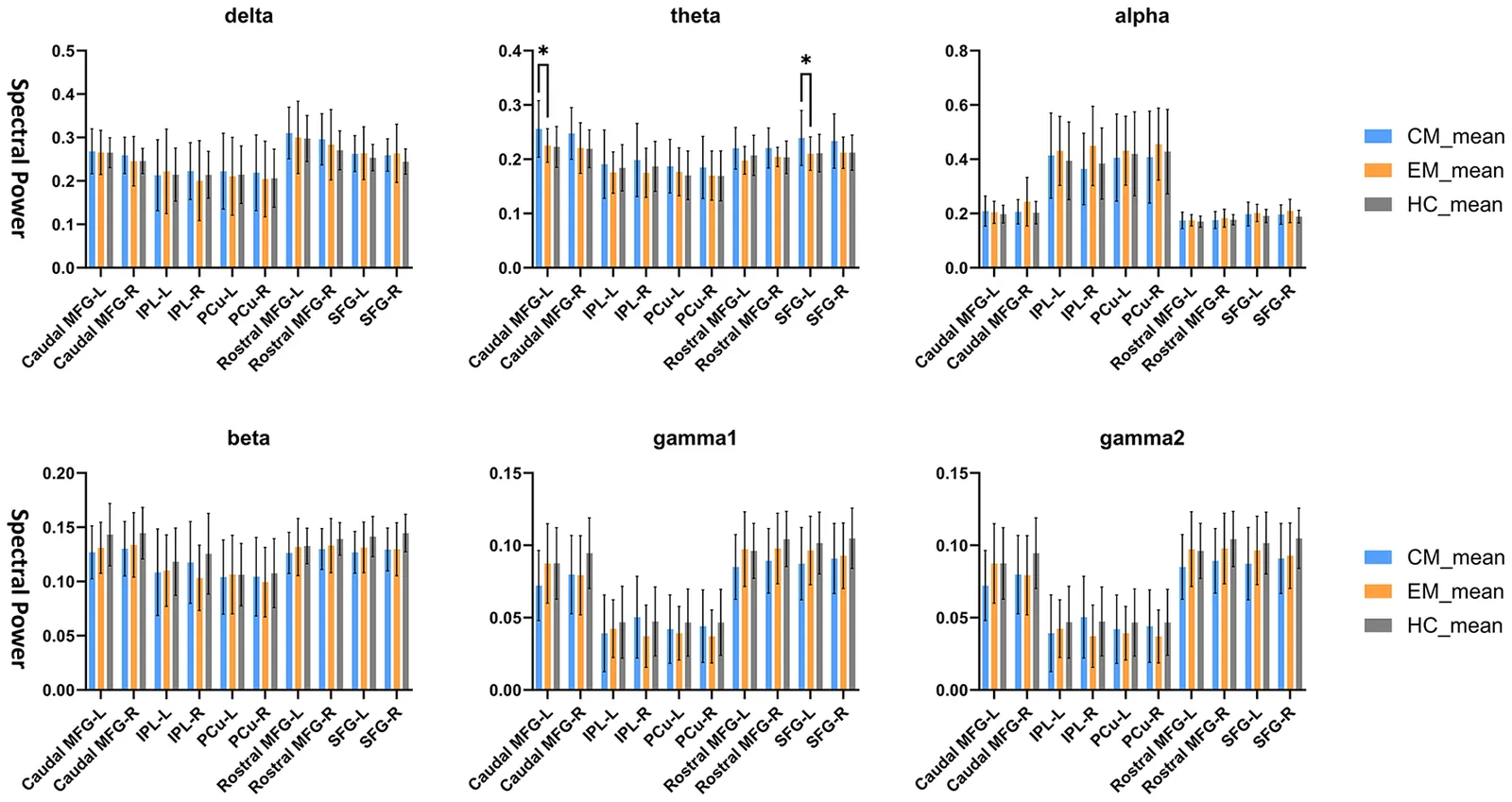
Comparison of the relative spectral power of the CM, EM, and HC groups. Values with ^*^ indicate significant differences only in the direct CM-EM comparison (Caudal MFG-L, *p* = 0.0106; SFG-L, *p* = 0.0122). Other between-group comparison results are reported in the main text and are not marked in the figure. The Bonferroni-corrected significance threshold was *p* < 0.0167. Bars represent the mean ± SD.

Group comparisons of resting-state relative PSD across the 10 CEN-related ROIs and six frequency bands showed that significant differences were mainly concentrated in the theta, beta, and gamma1 bands and were largely driven by the CM group (Figure 1). The covariate-adjusted ANCOVA identified four significant ROI-frequency combinations.

In the theta band, the CM group showed increased relative power. Specifically, relative power in the Caudal MFG-L was significantly higher in the CM group than in the HC group (*p* = 0.0082) and the EM group (*p* = 0.0106). A similar pattern was observed in the SFG-L, where the CM group also showed significantly higher relative power than both the HC group (*p* = 0.0086) and the EM group (*p* = 0.0122).

In contrast, the CM group showed reduced local relative power in the beta and gamma1 bands. In the beta band, relative power in the SFG-R was significantly lower in the CM group than in the HC group (*p* = 0.0089). In the gamma1 band, relative power in the Rostral MFG-R was also significantly lower in the CM group than in the HC group (*p* = 0.0106). No significant differences were observed in relative PSD between the EM and HC groups in any tested ROI or frequency band. In addition, no significant group differences were found in the delta, alpha, or gamma2 bands.

### 3.3 Functional connectivity analysis

**Figure 2:**
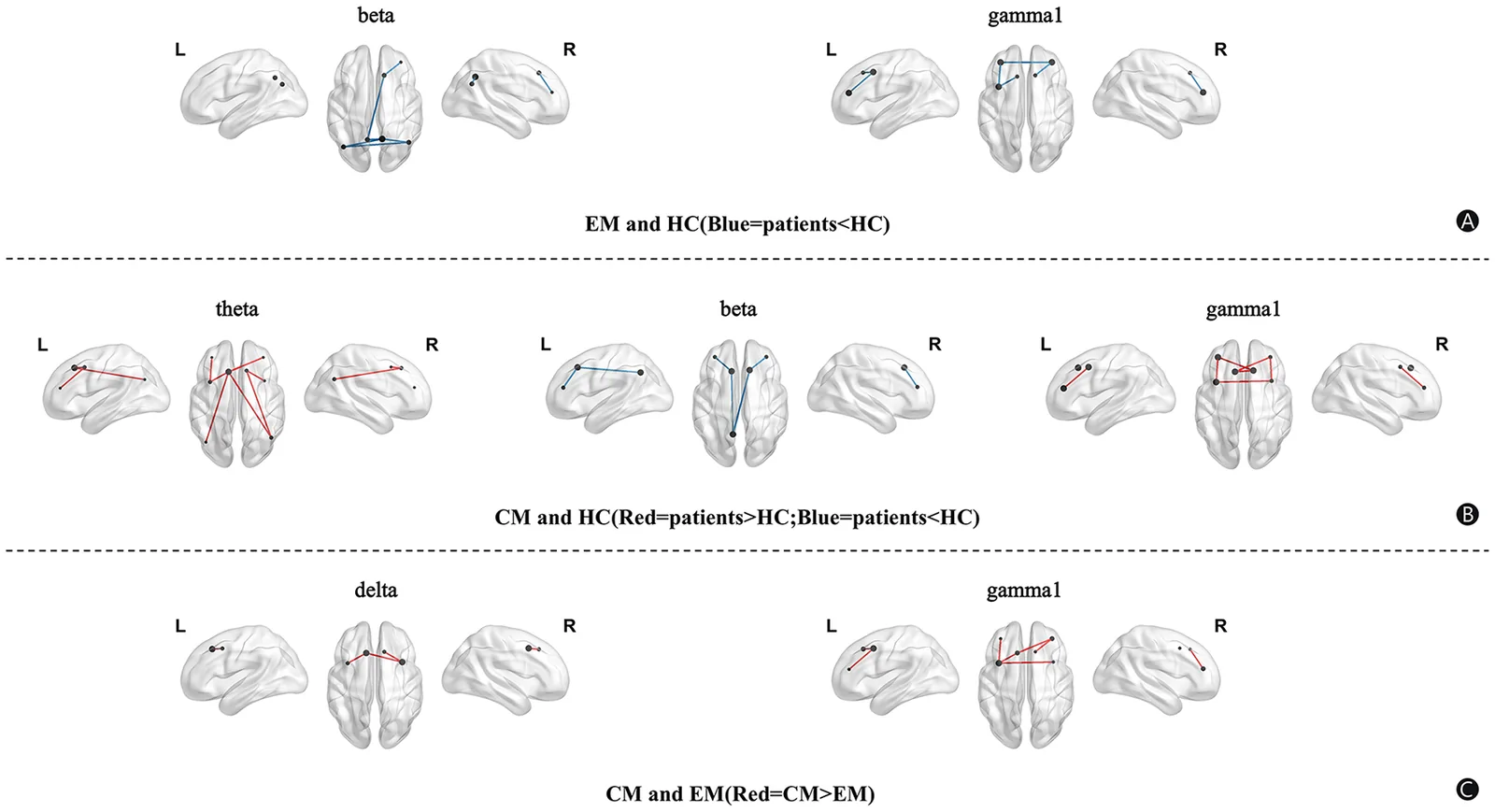
Group differences in CEN-related functional connectivity. The figure displays significant subnetworks identified by NBS across different frequency bands at an initial threshold of *p* < 0.001. The comparisons shown are between: **(A)** EM and HC, **(B)** CM and HC, and **(C)** CM and EM. Red lines indicate increased connectivity, and blue lines indicate decreased connectivity.

NBS analysis revealed marked frequency-specific group differences in functional connectivity among the 10 CEN-related ROIs. Because the connectivity changes identified at the initial threshold of *p* < 0.01 were relatively extensive, only significant sub networks identified at the more stringent initial threshold of *p* < 0.001 are reported below and illustrated in Figure 2. All reported sub networks survived 5,000 permutation tests and were significant at the component level after FWER correction. Overall, significant connectivity alterations were mainly observed in the theta, beta, delta, and gamma1 bands, whereas no significant group differences were found in the alpha or gamma2 bands.

#### 3.3.1 CM vs. HCs

Compared with the HC group, the CM group showed significant subnetworks in the theta, beta, and gamma1 bands.

In the theta band, the CM group showed more widespread connectivity increases. The significant sub network involved 8 nodes and 7 edges, mainly distributed across bilateral frontal regions and the inferior parietal lobule. The altered connections included Caudal MFG-L–Rostral MFG-L, Caudal MFG-L–SFG-L, IPL-L–SFG-L, IPL-R–SFG-L, Rostral MFG-R–SFG-L, Caudal MFG-R–SFG-R, and IPL-R–SFG-R.

In the beta band, the CM group showed reduced connectivity. The significant sub network involved 5 nodes and 4 edges, including PCu-L–SFG-L, Rostral MFG-L–SFG-L, PCu-L–SFG-R, and Rostral MFG-R–SFG-R, mainly involving connections between the precuneus and frontal regions.

In the gamma1 band, the CM group again showed increased connectivity, predominantly within frontal regions. The significant sub network involved 6 nodes and 6 edges, including Caudal MFG-L–Caudal MFG-R, Caudal MFG-L–Rostral MFG-L, Caudal MFG-R–Rostral MFG-R, Rostral MFG-R–SFG-L, Rostral MFG-L–SFG-R, and SFG-L–SFG-R.

#### 3.3.2 EM vs. HCs

Compared with the HC group, connectivity abnormalities in the EM group were relatively limited, with significant sub networks identified only in the beta and gamma1 bands.

In the beta band, the EM group showed reduced connectivity. The significant sub network involved 6 nodes and 6 edges, including IPL-L–IPL-R, IPL-L–P Cu-R, IPL-R–P Cu-R, P Cu-L–P Cu-R, P Cu-L–SFG-R, and Rostral MFG-R–SFG-R. This sub network mainly involved the bilateral inferior parietal lobules, precuneus, and right frontal regions.

In the gamma1 band, the EM group also showed reduced connectivity. The significant sub network involved 5 nodes and 4 edges, including Caudal MFG-L–Rostral MFG-L, Rostral MFG-L–Rostral MFG-R, Caudal MFG-L–SFG-L, and Rostral MFG-R–SFG-R. This subnetwork mainly involved the left Caudal MFG, bilateral Rostral MFG, and bilateral SFG.

#### 3.3.3 CM vs. EM

Direct comparison between the CM and EM groups showed significant connectivity differences in the delta and gamma1 bands, both characterized by stronger connectivity in the CM group than in the EM group.

In the delta band, the CM group showed an enhanced functional sub network involving 4 nodes and 3 edges, including Caudal MFG-L–SFG-L, Caudal MFG-R–SFG-L, and Caudal MFG-R–SFG-R. These changes mainly involved connections between the Caudal MFG and bilateral SFG.

In the gamma1 band, the significant sub network involved 6 nodes and 5 edges, including Caudal MFG-L–Caudal MFG-R, Caudal MFG-L–Rostral MFG-L, Caudal MFG-L–SFG-L, Rostral MFG-R–SFG-L, and Rostral MFG-R–SFG-R. Overall, this pattern was again characterized by enhanced connectivity predominantly within bilateral frontal regions.

### 3.4 SVM classification analysis

**Figure 3:**
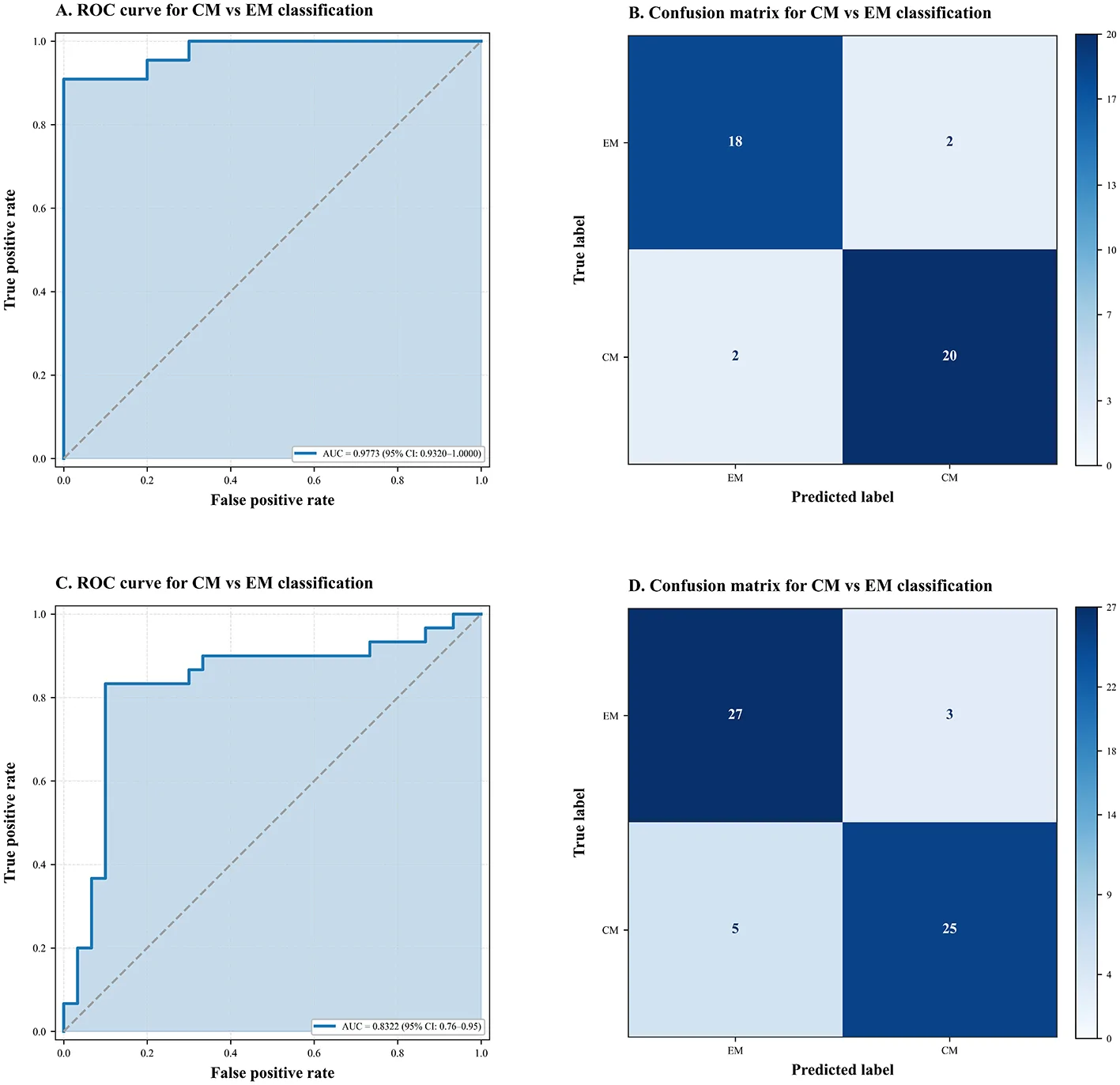
SVM classification performance for distinguishing CM from EM. **(A, B)** ROC curve and confusion matrix based on five-fold cross-validation in the original cohort. **(C, D)** ROC curve and confusion matrix obtained in the independent validation cohort.

To further evaluate the potential discriminative value of the CEN-related neurophysiological measures for CM and EM, an exploratory classification analysis was performed using a linear-kernel SVM classifier. The model was based on the 10 features listed in Table 1, including two theta-band PSD features and eight functional connectivity features. In the original cohort, five-fold stratified cross-validation yielded an accuracy of 90.48%, an AUC of 0.9773 (95% CI: 0.93–1.00), a sensitivity of 90.91%, and a specificity of 90.00% (Figures 3A, B). The finalized classifier was subsequently applied to an independent validation cohort comprising 30 patients with CM and 30 patients with EM. In the independent validation cohort, the classifier achieved an accuracy of 86.67%, an AUC of 0.8322 (95% CI: 0.76–0.95), a sensitivity of 83.33%, and a specificity of 90.00% (Figures 3C, D). These findings indicate that the classifier retained discriminative performance in the independent validation cohort.

## 4 Discussion

The present study showed that, during the interictal period, patients with CM exhibited more extensive CEN-related neuromagnetic abnormalities than patients with EM. These abnormalities showed a clear frequency dependence: rather than being diffusely distributed, they were mainly concentrated within the prefrontal–front oparietal network. Spectral abnormalities were limited to the theta, beta, and gamma1 bands and were largely driven by the CM group. Functional connectivity analysis revealed an even clearer gradient pattern: compared with both the HC and EM groups, the CM group showed broader abnormalities, whereas the EM group exhibited only more limited changes. Importantly, direct comparison between CM and EM further localized the between-subtype differences to enhanced prefrontal-dominant functional connectivity in the delta and gamma1 bands. Taken together, these findings suggest that migraine chronification is not merely characterized by a quantitative increase in headache burden, but is associated with more extensive alterations in executive-control-related cortical dynamics.

### 4.1 Spectral power alterations

One notable feature of the present findings is that the spectral abnormalities were selective rather than global. The CM group showed increased relative power in the theta band in the left caudal middle frontal gyrus and left superior frontal gyrus, whereas reduced relative power was observed in the beta band in the right superior frontal gyrus and in the gamma1 band in the right rostral middle frontal gyrus. This pattern does not support a generalized shift in oscillatory activity across all frequency bands. Instead, it suggests frequency-specific dysregulation within prefrontal CEN-related regions, characterized by enhanced low-frequency oscillations and attenuated high-frequency oscillations. This interpretation is broadly consistent with the recent resting-state MEG/EEG meta-analysis by Zebhauser et al. (2024) which identified increased interictal theta activity and reduced alpha/beta connectivity as the most reproducible electrophysiological findings in migraine. In this context, the frontal theta enhancement observed in the present study may reflect reduced efficiency of control-related processing or sustained compensatory recruitment in the context of recurrent headache burden, whereas reduced beta/gamma activity in the present study may suggest impaired fast oscillatory coordination related to top-down regulation and cortical integration (Lim et al., 2016; Puledda et al., 2023; Chertic et al., 2025). Nevertheless, these interpretations should be made with caution, because resting-state oscillatory findings are not task-specific and may reflect the combined influence of chronic pain, attentional bias, altered cortical excitability, and compensatory processes.

### 4.2 Functional connectivity alterations

The functional connectivity results extend this interpretation from local oscillatory abnormalities to network-level reorganization. Compared with HCs, the CM group showed increased theta-band connectivity involving bilateral prefrontal regions and the inferior parietal lobules, reduced beta-band connectivity centered on precuneus–prefrontal connections, and increased gamma1-band connectivity mainly among bilateral frontal nodes. In contrast, the EM group exhibited only more limited reductions in beta- and gamma1-band sub networks. The coexistence of hyper connectivity and hypo connectivity is itself noteworthy, suggesting that information transfer within executive-control-related circuits is not uniformly increased or decreased during migraine chronification, but instead appears to be redistributed across the network. This view is consistent with a large body of neuroimaging evidence indicating that CM is characterized by large-scale brain network reorganization rather than isolated dysfunction of a single pain-transmission pathway (Androulakis et al., 2017; Schulte and May, 2017; Coppola et al., 2020; Zou et al., 2021).

The direct comparison between CM and EM is particularly important, because it indicates that the present findings do not merely reflect the general distinction between migraine and healthy controls. In this study, the CM group showed significantly stronger connectivity than the EM group in the delta and gamma1 bands, with the differences mainly involving connections between the middle frontal gyri and superior frontal gyri. These findings suggest that the transition from episodic to chronic migraine is not simply an amplification of abnormalities already present in EM, but rather involves altered coupling patterns within prefrontal executive-control-related circuits (Nieboer et al., 2020; Planchuelo-Gómez et al., 2020; Hsiao et al., 2022). In this context, increased connectivity should not be interpreted as indicating more efficient control function. Instead, it may reflect altered network organization rather than enhanced efficiency, possibly indicating inefficient information processing or compensatory engagement within prefrontal executive-control-related circuits (Kucyi et al., 2014; Nieboer et al., 2020). Recent studies further support this interpretation by showing that migraine-related cognitive dysfunction, particularly during the interictal period, is closely associated with distributed brain network abnormalities centered on prefrontal systems rather than being solely attributable to sensory dysfunction (Lee et al., 2021; Chen et al., 2024; Fernandes et al., 2024).

Another point worth emphasizing is the clear anatomical clustering of the observed abnormalities. Although the analysis included 10 predefined CEN-related regions, the most prominent effects were concentrated in prefrontal and front oparietal nodes rather than being evenly distributed across all ROIs. This spatial clustering is meaningful. It suggests that migraine chronification preferentially affects brain regions associated with attentional allocation, executive-control-related processing, and top-down regulation (Lorenz et al., 2003; Seminowicz and Moayedi, 2017; Urien and Wang, 2019). In other words, the abnormal pattern in CM appears organized rather than diffuse. This may also help explain why the EM group showed only limited abnormalities relative to HCs: if chronification reflects progressive brain network reorganization, then more widespread impairment of prefrontal control-related circuits would be expected to emerge more clearly in CM (Hashmi et al., 2013; Schulte and May, 2017; Hsiao et al., 2022). This interpretation is also compatible with previous MEG studies linking migraine chronification to altered beta-band connectivity within pain-related cortical regions (Hsiao et al., 2021).

Frontal theta- and delta-band abnormalities are not necessarily specific to isolated CEN dysfunction, as low-frequency activity may also be influenced by chronic pain and broader cognitive-affective processes (Fernandes et al., 2024; Chertic et al., 2025). In the present study, the observed group differences remained significant after adjustment for HAMA and HAMD scores, indicating that they were not fully attributable to measured anxiety and depressive symptoms. However, residual confounding and contributions from other cognitive-affective processes cannot be excluded. These findings therefore reflect alterations involving CEN-related regions and should not be interpreted as evidence that CEN dysfunction causes emotional symptoms.

### 4.3 Classification value

The exploratory SVM analysis adds preliminary translational value to the present findings, but its interpretation should remain conservative. Based on two theta-band PSD features and eight functional connections that differed between the CM and EM groups, the classifier achieved relatively high cross-validated performance in distinguishing the two migraine subtypes. This suggests that combining local oscillatory information with network-level coupling features may capture clinically meaningful differences more effectively than analyses focusing on a single level alone (Hsiao et al., 2022, 2023). However, this result should be regarded only as proof of concept and not as evidence of a mature biomarker. Recent methodological recommendations in migraine-related machine-learning research have emphasized that classification performance should be interpreted in the context of phenotypic homogeneity, sample size, quality control, transparency of data splitting and feature selection, interpretability, and external validation (Varoquaux et al., 2017; Petrušić et al., 2024, 2025). Importantly, the classifier retained good discriminative performance in the independent validation cohort, with an accuracy of 86.67% and an AUC of 0.8322. Nevertheless, because the validation cohort was obtained from the same center, the classifier should still be considered exploratory until further validated in larger multicenter and externally acquired datasets.

From a broader clinical perspective, the present findings support the view that CM and EM are not neuro physiologically equivalent states (Schwedt et al., 2015; Planchuelo-Gómez et al., 2020; Hsiao et al., 2022). The predominance of prefrontal and front oparietal abnormalities in CM suggests that executive-control-related circuits may provide a useful systems-level window into the mechanisms of migraine chronification (Androulakis et al., 2017; Seminowicz and Moayedi, 2017). This does not imply that the CEN alone can explain CM. Rather, it suggests that dysfunction of brain networks involved in cognitive control and pain regulation constitutes an important component of the chronic migraine state. In this sense, the present study further adds to the literature supporting the view that CM should be understood as a disorder of distributed brain network dysfunction, with executive-control-related abnormalities representing one part of its broader pathophysiological architecture.

### 4.4 Limitations

Several limitations should be considered when interpreting the present findings. Because this was a cross-sectional study, no causal conclusions can be drawn regarding the relationships among CEN-related abnormalities, migraine chronification, and emotional symptoms. We are currently addressing this question through longitudinal follow-up to further clarify the temporal relationships and associations among these factors. In addition, the CM group had a greater clinical burden and more frequent analgesic use than the EM group. Although these differences are clinically expected in CM, they may have partly influenced the observed resting-state MEG abnormalities and therefore represent potential confounding factors. The sample size was also modest, which is a common limitation in MEG research and may have reduced the stability of some subgroup-specific findings; replication in larger cohorts and independent samples is needed. The statistical framework also retained an exploratory element. Although the analyses were hypothesis-driven and restricted to predefined CEN-related regions and frequency bands, and Bonferroni correction was applied to pairwise comparisons within each ROI–frequency measure, we did not perform a more stringent global correction across all ROI–frequency combinations. This approach may have helped avoid missing potentially meaningful effects, but it also leaves open a greater risk of false-positive results. For this reason, the present findings should be viewed as preliminary until they are confirmed in independent datasets using stricter correction procedures. In addition, the analysis was limited to 10 predefined CEN-related regions and therefore could not comprehensively characterize whole-brain network interactions. Studies with broader network coverage and more complete network-level analyses may help clarify how the CEN interacts with other large-scale brain networks. Furthermore, standardized cognitive and executive-function assessments were not collected, which limits our ability to directly relate the observed CEN-related neuromagnetic abnormalities to cognitive or executive performance. Future studies incorporating such assessments will be needed to further clarify the functional significance of these findings. Finally, although the exploratory SVM model was evaluated in an independent validation cohort, external validation has not yet been performed. Further testing in larger multicenter and external validation datasets will be required to determine its broader generalizability.

## 5 Conclusion

This study demonstrated that CM is associated with more extensive and frequency-specific CEN-related neuromagnetic abnormalities than EM, particularly within prefrontal–front oparietal circuits. These findings suggest that migraine chronification involves altered executive-control-related cortical dynamics during the interictal state. Furthermore, combined CEN-related spectral and connectivity features may have potential value for differentiating CM from EM.

## Data Availability

The datasets generated and/or analyzed during the current study are not publicly available. Anonymized data can be obtained from the corresponding author upon appropriate request.
