# Supplementary material for: Central executive network-related neuromagnetic abnormalities in chronic and episodic migraine: a resting-state magnetoencephalography study
Source: Front Neurosci. 2026 Sep 1;20:1865928. doi: 10.3389/fnins.2026.1865928 (PMC13574956; doi:10.3389/fnins.2026.1865928)
Supplement: Supplementary file 1 [file Table_1.docx]

**Table S1. Demographic and clinical characteristics of the independent validation cohort**

| Clinical characteristics | CM | EM | p-value |
| --- | --- | --- | --- |
| Sex | 15 M/15 F | 15 M/15 F | 1.000 |
| Age(y) | 42.4 ± 8.1 | 40.2 ± 9.9 | 0.350 |
| Headache frequency (days/month) | 18.8 ± 8.2 | 6.6 ± 3.7 | <0.001* |
| Disease duration (y) | 16.9 ± 8.6 | 14.9 ± 8.7 | 0.374 |
| Painkiller use (days/month) | 6.6 ± 5.9 | 3.3 ± 2.1 | 0.007* |
| VAS score  HAMA score  HAMD score | 7.7 ± 1.6  8.4 ± 4.2  6.9 ± 3.5 | 6.6 ± 1.6  8.3 ± 3.8  5.7 ± 3.2 | 0.010*  0.923  0.171 |

SD, standard deviation; CM, chronic migraine; EM, episodic migraine; HAMA, Hamilton Anxiety Rating Scale; HAMD, Hamilton Depression Rating Scale; VAS, Visual Analogue Scale; M, male; F, female. *p < 0.05 is statistically significant.
